# Experience of participation in a WhatsApp support group for empowering newly graduated nurses during transition in the Western Cape, South Africa

**DOI:** 10.4102/curationis.v49i1.2844

**Published:** 2026-06-03

**Authors:** Rita O. Abiodun, Felicity Daniels, Jennifer A. Chipps, Christoph Pimmer

**Affiliations:** 1 School of Nursing, Faculty of Community Health Sciences, University of the Western Cape, Cape Town, South Africa; 2 School of Business, University of Applied Sciences and Arts, Aargau, Switzerland

**Keywords:** newly-graduated-nurses, nursing-education, study-to-work, transition, WhatsApp

## Abstract

**Background:**

Transitioning from academic study to professional practice is a critical and often challenging phase for newly graduated nurses. Providing structured support during this period is essential for fostering confidence, competence and professional growth.

**Objectives:**

This study aimed to explore the experiences of new nurse graduates from a university in the Western Cape following their participation in a WhatsApp peer support group designed to facilitate their transition to practice.

**Method:**

A qualitative, exploratory descriptive design was used. A 12-week WhatsApp-based intervention was implemented, with weekly discussions on predetermined topics related to professional development. Twelve participants took part in in-depth interviews after the intervention – four through an open call and eight through individual invitations – until data saturation was reached. Data were collected using an interview guide and were analysed thematically.

**Results:**

Four major themes emerged: (1) information sharing empowered participants and enhanced professional development; (2) a supportive community of practice was formed; (3) the online presence of moderators fostered engagement; and (4) participants experienced personal growth through skill development and relevant discussions.

**Conclusion:**

The study demonstrated that a structured online peer support intervention can effectively promote empowerment, professional confidence and collaboration among newly graduated nurses.

**Contribution:**

This research contributes to the growing body of evidence on the use of digital platforms – specifically WhatsApp – for facilitating professional transitions and continuous learning. It provides practical insights for designing supportive, technology-enhanced learning environments in nursing education and practice.

## Introduction

The transition to a professional nursing role presents a considerable challenge for newly graduated nurses, even when structured transition programmes are in place (Nour & Williams 2019; Tawash, Cowman & Anwar 2024). Transition involves a series of changes in roles, responsibilities and goals, and is rooted in the broader philosophy of change and development (Schumacher & Meleis 1994). According to Schumacher and Meleis, the impact of a transition on an individual’s well-being, such as experiences of stress, discomfort or professional uncertainty, depends on how effectively the components of the transition align with the objectives of the change process. In the context of nursing, well-structured transition programmes can facilitate this alignment, helping newly graduated nurses navigate role adaptation, reduce adverse experiences and achieve professional competence.

In nursing, targeted interventions, including structured transition programmes and peer-learning initiatives, can support this alignment by facilitating role adaptation, enhancing professional confidence and reducing negative experiences during the transition period (Charette et al. 2023; Raqueno et al. 2025).

An intervention was developed to support and empower new graduates during their transition year from university to the workforce. The acronym mLEAP was coined to represent what the programme stands for – Mobile Intervention to Link, Empower and Professionalise new graduate nurses.

This programme was developed by reviewing the support needs of new graduate nurses. According to this study, Newly Graduated Nurses are nurses with no or less than 1 year of clinical practice experience, not including nurses transitioning between specialities. Kanter’s theory of structural empowerment was used as a framework for this intervention. This is an organisational theory that focuses on contextual factors within organisations that promote healthy working environments for individuals (Kanter 1977). The theory has four important structures that individuals need access to for empowerment: (1) opportunity to grow and advance within the organisation; (2) information regarding the work; (3) support from colleagues and leaders; and (4) resources (Kanter 1977). It was envisaged that this framework would guide the development and implementation of the support through access to information and resources, and autonomous supportive discussions via informal alliances (Kusurkar 2019). Participants would link with relevant peers, leaders, and moderators for empowerment during the transition year through access to opportunities to grow, and access to information, support and resources via an informal alliance using an Instant Messaging application (WhatsApp).

The identified needs were presented by the doctoral student and ranked by a group of stakeholders using a nominal group technique workshop. Mobile Intervention to Link, Empower and Professionalise is a moderated 12-week professional education transition programme of peer support. Each of the 12 weeks of the intervention was unique, with a new topic for discussion each week. The participants’ interactive responses to the topic were influenced by their personal experiences or observations in their respective workplaces. The pedagogical approach followed can be described as one of conversational learning with integrated elements of reflective practice (Li et al. 2024; Ro & Ishino 2025). In addition to the topics, the researcher posted scenarios related to a particular nursing situation and asked participants’ opinions on the scenarios. Moreover, at the end of each session, the researcher posted resources related to the topic, and participants could engage. As a result of the geographic spread of participants, the programme was delivered digitally via WhatsApp. WhatsApp was chosen over Learning Management System (LMS) platforms like Moodle or Blackboard because it is widely accessible, familiar, low-cost in terms of data and supports real-time interaction and push notifications, which can enhance engagement and peer learning (Lee, Casillas-Martín & Suárez-Lantarón 2023).

To evaluate the implementation of this intervention, the following research question was posed: What are the experiences of new nurse graduates from a university in the Western Cape participating in a WhatsApp peer transition support group?

## Literature review

### Nurses’ general need for support

The process of nurse transition holds significant importance in ensuring the delivery of high-quality healthcare services to patients and addressing the global issue of nurse retention (World Health Organization 2020).

The transition of newly graduated nurses into professional practice is a critical period that significantly influences the delivery of high-quality healthcare and nurse retention globally. During this phase, nurses are particularly vulnerable to social isolation and workplace pressures, which can contribute to stress, reality shock and an increased risk of leaving the profession (Edwards et al. 2015; Gardiner & Sheen 2016; Nour & Williams 2019; Walker, Douglas & Chung 2017). A lack of sufficient social and emotional support has been consistently identified as a key factor driving occupational stress and burnout among novice nurses (Kakemam et al. 2019). Inadequate support may lead to the exhaustion and subsequent departure of novice nurses from the profession.

### Support possibilities

Literature emphasises numerous advantages of peer support and mentorship in the nursing profession, including the reduction of stress and professional isolation, improved nurse retention rates, and enhanced quality of care (Jenkins et al. 2021). The phenomenon of newly graduated nurses engaging in interpersonal interactions, such as meetings, socialising and exchanging experiences, has been reported to contribute to the enhancement of their collective capacity to manage stress (Pålsson et al. 2021). Peer learning is a collaborative educational approach wherein individuals who share a common context engage in the process of learning together by actively interacting with one another (Sarmin, Khatun & Islam 2025). Evidence indicates that structured peer support interventions positively influence the transition of newly graduated nurses by enhancing emotional resilience, reducing feelings of isolation, and improving readiness for autonomous practice; for instance, a systematic review found that virtual peer support, togetherness in practice, and paired readiness-building significantly improve new nurses’ well-being and job satisfaction (Jenkins et al. 2021). Qualitative studies further suggest that, although online peer groups hold promise for supporting Newly Graduated Nurses’ (NGNs) adjustment, their effectiveness depends on careful design and organisational backing to overcome participation barriers (Pålsson et al. 2022).

An alternative approach is the utilisation of problem-solving-oriented peer support groups as a potential means of mitigating stress and burnout among healthcare professionals. According to Othman et al. (2018), engagement in peer support groups has the potential to enhance individuals’ ability to manage stress, augment their level of contentment with their occupation, and mitigate the occurrence of burnout and psychological anguish within the healthcare sector. The problem-solving methodology employed within these groups facilitates the generation of pragmatic solutions to work-related obstacles, fostering self-control and empowerment, ultimately resulting in a decrease in stress levels and mitigating the risk of burnout (Ausserhofer et al. 2014). It is expected that these peer support groups would create an environment where nurses can experience emotional and social support, feel valued, and be motivated to engage in collaborative problem-solving with their peers to address workplace challenges. Moreover, the significance of proficient socialisation of newly hired employees in mitigating the inclination to resign, reducing employee turnover, alleviating tension and preventing burnout is noteworthy (Vilayil 2021).

In addition, peer support groups can function as informal mentoring, offering guidance and support to junior healthcare workers (Lim et al. 2022). Despite the recognised benefits of peer support, traditional approaches are often limited by geographic dispersion, shift schedules and resource constraints, particularly in the South African public health sector. This underscores the need for innovative, accessible interventions that extend beyond informal chat groups. A theoretically informed digital mentorship model, grounded in Kanter’s framework of empowerment and support, can bridge this gap by providing structured, evidence-based guidance, facilitating professional confidence, and fostering connectedness among newly graduated nurses (Evans 2025). Exploring the feasibility of delivering such an intervention via WhatsApp addresses both contextual constraints – such as limited data access and dispersed healthcare facilities – and the urgent need to strengthen transition support, making it highly relevant for the current South African healthcare context (Abiodun et al. 2019; Guillaume et al. 2023). Nevertheless, it is imperative to acknowledge certain potential obstacles that may arise during the implementation of peer support groups within healthcare settings. For example, certain individuals may experience discomfort when engaging in conversations about personal matters with their peers or may exhibit reluctance in seeking assistance because of concerns of societal disapproval or criticism (Agarwal, Brooks & Greenberg 2020).

### Technology as a means for transition support

Mobile technology is a widely accessible means of communication. The healthcare industry has witnessed a surge in the utilisation of technology for communication, training, seminars and support groups among novice nurses. Digital technologies facilitate convenient contact and information sharing within the nursing profession (Conte et al. 2023). These technologies facilitate remote communication, collaboration and knowledge dissemination, hence enhancing accessibility and flexibility in the learning and working environments. For example, in a study conducted by Chipps et al. (2015), it was found that social media platforms such as Facebook and WhatsApp have shown to be effective tools for providing support in rural midwifery practice in South Africa. Social media was also found to promote a sense of community and connection outside of work. Newly graduated nurses need a collaborative learning environment to encourage critical thinking and share best practices. Melissant et al. (2024) recognised the dearth of research on the utilisation of technology for supporting nurses’ transition and further noted that the few studies in this area are lacking in terms of theoretical underpinnings.

## Research methods and design

### Intervention design and development

The transition support intervention was designed as a structured, theory-informed digital peer support programme to support newly graduated nurses during their community service (transition) year. The development of the intervention was guided by Kanter’s Theory of Structural Empowerment, which emphasises access to information, support, resources and opportunities as critical to professional growth and role transition. This framework informed both the selection of content and the interactive design of the intervention.

Intervention content was informed by two key sources. Firstly, a comprehensive review of published literature examining the experiences and needs of newly graduated nurses during their community service year in South Africa (Abiodun et al. 2019) was conducted to identify common transition challenges and support gaps. Secondly, a needs assessment was undertaken during the first week of a pilot study, allowing participants to identify priority areas requiring support. Findings from these sources informed topic selection and ensured the relevance and contextual appropriateness of the intervention. Based on the identified needs, weekly topics were developed, and scenario-based prompts were created to facilitate reflection, discussion and experiential learning.

### Intervention delivery

The intervention was delivered via a WhatsApp instant messaging group, selected for its accessibility, familiarity and low data requirements. The group was established and managed by the principal researcher, a PhD student, and included a total of 54 participants. The programme was conducted over several weeks, with one topic addressed per week, as outlined in Table 1.

**TABLE 1:** Outline of Mobile Intervention to Link, Empower and Professionalise transition programme.

Week	Topic discussed
Week 1	Welcome and pre-survey
Week 2	Needs assessment and programme orientation
Week 3	Scope of practice
Week 4	Inter-professional relationship
Week 5	Professional and ethical standards
Week 6	Ethical decision making
Week 7	Professional accountability
Week 8	Conflict management
Week 9	Stress management and self-care
Week 10	Career management
Week 11	Conclusion and post-survey
Week 12	Post survey follow up

Each weekly session followed a consistent structure. Topics were introduced using a short animated video or scenario designed to stimulate reflection and engagement. This was followed by a moderated group discussion scheduled at times agreed upon by participants to maximise participation. To accommodate varying work schedules, a follow-up discussion was held the following day for participants unable to participate in the initial session. At the end of each week, key discussion points and insights were summarised during a dedicated session. A final session then provided participants with relevant published evidence, additional resources and useful links related to the weekly topic.

The programme was facilitated by two moderators: the principal researcher, trained in qualitative research and digital learning facilitation, and a Nurse Educator with experience in clinical education and mentorship. To minimise potential researcher bias associated with the dual role of facilitator and researcher, discussions were guided using a predefined facilitation framework that employed standardised, non-directive prompts.

### Participant procedures

Participants were enrolled in the intervention group following recruitment and informed consent procedures. Upon joining the WhatsApp group, participants were provided with clear rules of engagement, including expectations for respectful communication, confidentiality and voluntary participation. These guidelines were outlined at the start of the programme to promote a safe and supportive learning environment.

Participants were invited to contribute by sharing experiences, reflections and opinions related to the weekly topics; however, participation was not mandatory, and members could engage at their own pace. Various scenarios aligned with weekly topics were shared within the group to support discussion and reflective learning. Participants assigned to the control group did not receive any structured transition support during the study period.

### Design and sample

A qualitative exploratory, descriptive design was used to obtain recorded semi-structured interview data from 12 participants. Maximum variation purposive sampling was employed to select 12 participants from the 54 intervention recipients, ensuring diversity in characteristics such as participants who engaged more and participants with less engagement to capture different perspectives.

### Data collection

Telephonic in-depth interviews were conducted by the primary researcher (PhD student) in English between May and July 2019. Qualitative interviews were conducted with a sub-sample of the intervention group (*n* = 12). The interview was guided by using an interview guide with probes that elicited deeper discussion. Participants were asked a broad, open-ended question: *Please describe your experiences of participating in the WhatsApp professional support group, with specific reference to your usage, perceived benefits, and connectivity*. Probing questions were subsequently employed to elicit further detail and clarification. Each interview lasted about 30–45 min and was recorded verbatim. Data was collected until data saturation. Data saturation was achieved during the later stage of data collection and analysis, whereby no new themes or insights emerged from additional participant contributions. Sampling was purposeful to ensure diverse perspectives relevant to the research question. Throughout the study, emerging categories and themes were continuously monitored, and redundancy in participant responses indicated that saturation had been reached, confirming the adequacy of the data for robust qualitative analysis.

### Analysis

The data collected were analysed thematically. The interview recordings were transcribed verbatim and were read and re-read to achieve immersion. Subsequently, all Microsoft Word transcripts were imported to ATLAS.ti version 1.6 (ATLAS.ti GmbH, Berlin, Germany) for organisation and analysis. Codes were generated that revealed the experience of the participants in the WhatsApp groups in the specific context. Similar or related codes were further abstracted into meaningful categories and finally grouped into the main themes (Braun & Clarke 2016). Thematic analysis was done using the inductive analytical approach to make sense of the information gathered following interpretive approaches.

### Methodological rigour

The study design adheres to established standards of methodological rigour, including transparency, reproducibility and ethical integrity, to ensure the reliability and validity of the findings. The principal investigator, a PhD student with clinical nursing experience and a background in teaching medical–surgical nursing and research methodology, contributed both practical and scholarly expertise, supporting rigorous data collection, thoughtful analysis and credible interpretation of the findings.

An intentional analytic decision was made not to merge categories that appeared to overlap. This was done to preserve the nuance in participants’ accounts and ensure that distinct aspects of their experiences did not collapse.

### Trustworthiness

Trustworthiness was established throughout the research process. The study’s credibility was established through the utilisation of researcher triangulation. Transferability was ensured by providing a comprehensive description and participant exemplars. Dependability was established by maintaining meticulous and well-documented records. Lastly, conformability was achieved through consensus reached among the researcher and supervisors via discussions. The attainment of researcher reflexivity was accomplished by the meticulous documentation of study decisions and researcher debriefings throughout the research.

### Ethical considerations

Ethical approval for the study was obtained from the Biomedical Research Ethics Committee at the University of the Western Cape (Reference: BM18/4/12). Permission to conduct the study was also granted by both the University and the Department of Health. All participants provided written informed consent prior to the commencement of the study. Participation was entirely voluntary.

Participants were enrolled in the study prior to their graduation from nursing school. During the final year, the researcher conducted an enrolment survey in which nursing students were informed about the purpose and nature of the study. Those who agreed to participate signed a consent form and provided their phone numbers to facilitate post-graduation contact.

To ensure confidentiality and compliance with data protection standards, including the *Protection of Personal Information (POPI) Act*, several safeguards were implemented. The WhatsApp group used for the intervention was created and moderated by the PhD student (the primary researcher). All written discussions on the platform were conducted with prior consent from the participants, and strict group rules were established through consensus with the participants and regularly monitored by the moderators. These rules included prohibitions on sharing identifiable information about patients, colleagues or workplaces, maintaining respectful communication, and ensuring that discussions remained relevant to the learning topics. Compliance with the *POPI Act* guided the management, storage and sharing of all participant data throughout the study.

Although participants’ phone numbers were necessary for communication, personal identifiers were not included on any data collection tools. There was no call cost to participants, as the researcher made the calls. All surveys and content analysis were conducted anonymously using unique codes. Data forms and online submissions were anonymised and securely stored. The researcher ensured that any data collected would be stored for a minimum of 5 years following publication and subsequently uploaded to Kikapu, the university’s secure research data repository.

To minimise the potential for bias, the researcher maintained a neutral role throughout the intervention. The researcher refrained from private or individual communication with participants through WhatsApp and instead focused solely on facilitating the structured elements of the intervention. A nurse educator, who was also a member of the WhatsApp group, supported the delivery and moderation of the programme. However, it is acknowledged that complete neutrality is difficult to achieve, and the positive outcomes reported may have been influenced by participants’ familiarity with the researcher or their perception of being respected and supported.

A proactive plan was implemented to support ethical oversight during the intervention, which included active moderation of discussions, clear guidelines on professional conduct and confidentiality, informed consent procedures, and ongoing monitoring to prevent the sharing of identifiable patient information. Weekly check-in meetings with the researcher were conducted to address implementation challenges and participant concerns. Additionally, stakeholder roles within the mLEAP project were outlined as non-participatory, with responsibilities focused on monitoring for bias and ensuring ethical integrity throughout the study.

## Results

### Participant characteristics and intervention context

A total of 54 newly graduated nurses participated in the peer learning intervention. Data saturation was achieved through semi-structured interviews with 12 participants aged between 23 years and 45 years, who were allocated across various clinical units and when no new themes or sub-themes emerged from the interview. The professional transition period spanned 1 year, while the peer learning intervention commenced 2 months post-transition and continued for 12 weeks. This timing allowed participants to reflect meaningfully on their early transition experiences and the influence of the intervention during a critical adjustment phase.

Thematic analysis resulted in four overarching themes and 10 categories:

- **Theme 1:** Information sharing as a pathway to empowerment and professional growth.
- **Theme 2:** A community of practice where participants supported one another.
- **Theme 3:** Moderators as catalysts of structured support and access to resources.
- **Theme 4:** Empowerment through integration of personal development and contextual relevance.

Although these themes are presented as distinct, some overlap naturally occurred, reflecting the complex and interconnected nature of lived experiences, as described by Braun and Clarke (2016). For example, professional and social connectedness were closely related but remained separate to differentiate professional belonging from emotional support. Similarly, discussion topics grounded in real-life experiences and perceived relevance of topics were treated as distinct categories to capture different dimensions of learning and empowerment. Finally, some challenges were identified, and future improvements for the programme were suggested (Table 2).

**TABLE 2:** Themes and categories.

Theoretical framework	Themes	Categories
Access to information (data, technical knowledge, expertise required to function effectively in one’s position)	1. Information sharing as a pathway to empowerment and professional growth	1.1. Sharing of relevant information, materials and real-life experiences 1.2. Empowerment through engagement on a wide range of topics
Access to support (feedback and guidance received from superiors, peers and subordinates)	2. A community of practice was formed where participants supported each other	2.1. Professional connectedness 2.2. Social connectedness
Access to resources (time, material, money, supplies and equipment necessary to fulfil organisational goals)	3. Moderators as catalysts of structured support and resource access	3.1. Online presence of moderators 3.2. Moderator support and guidance on managing specific situations 3.3. Useful materials and knowledge shared
Opportunity to learn and grow (autonomy, growth, sense of challenge, and the chance to learn and grow)	4. Empowerment through integration of personal development and contextual relevance	4.1. Development of personal skills 4.2. Discussion topics included real-life experience 4.3. Relevance of the discussion topics

### Theme 1: Information sharing as a pathway to empowerment and professional growth

Theme 1 focuses on access to information on a wide range of topics relevant to transition and the sharing of real-life experiences. It illustrates how access to information and shared experiences created pathways for empowerment and professional identity development among newly graduated nurses. The WhatsApp group functioned not only as a forum for exchanging materials but also as a dynamic learning space where participants transformed shared knowledge into confidence and professional competence.

#### Sharing of relevant information, materials and experiences

One of the important benefits of this group was that participants derived advantages by engaging in the exchange of pertinent information, resources and practical experiences, hence fostering their professional growth. The information shared in the group was relevant to the current experiences of the participants, which proved to be beneficial.

This helps participants develop critical thinking and decision-making skills, and a deeper understanding of patient care. For instance, one of the participants stated that:

‘… it provided information and resourceful things that guides you when you come into contact with similar situations’ (Participant 8, 31 years, Female).

Similarly, another stated that:

‘… [*T*]he group really helped me, it … guided me to be a professional, and also to be a professional at work. It also gave me the attributes of a professional, how one can be at work.’ (Participant 9, 29 years, Female)

The topics discussed served as a refresher of what the participants learnt in the nursing programme, enabling them to enhance their communication and advocacy skills, and to effectively manage conflict and conduct themselves in a professional manner within the scope of practice:

‘… I think we were in the first week of our WhatsApp group … so the doctor told the nurse to do … and the nurse was not supposed to do … because she was performing out of his or her scope of practice.’ (Participant 3, 28 years, Female)

This peer interaction also helped individuals explore other ideological perspectives on topics, thereby boosting their understanding.

Furthermore, the newly graduated nurses had a sense of empowerment because of their active involvement in various discussions and deliberations, such as ethics, trauma management and the scope of their practice. Additionally, they acquired knowledge and skills on topics such as interpersonal relationships and record-keeping. Topics such as emotional intelligence, conflict resolution and insubordination were also regarded as advantageous. This was confirmed by a participant who stated:

‘… I think that the group is very helpful, sometimes we, some of us are not very interactive, but the students grabbed some information that we use in our working facilities … like sometimes we are gonna talk about trauma, gave me an insight of the scope of practice, the interpersonal relationships and those things which builds my professionalism.’ (Participant 4, 38 years, Male)

Another participant supported this by stating that:

‘… [*S*]o just how to communicate better, just like conflict management, it really taught me things that I would say is beneficial to us as nursing professionals.’ (Participant 6, 27 years, Female)

#### Empowerment through engagement on a wide range of topics

Another significant benefit of this WhatsApp support group was the opportunity for newly graduated nurses to engage with a wide range of topics. This exposure to diverse discussions, clinical scenarios and shared materials among peers created an environment conducive to learning and professional growth. The collective sharing of experiences and knowledge allowed new nurses to develop a broader understanding of nursing practices and situational responses across various settings. This was confirmed by Participant 6, 27 years, Female, who stated:

‘… I think I shared something about that and I said I have only had good experiences thus far with the difference topics we dicussed like advocacy, conflict and … scope of practice, that are very helpful where I am working, am able to ask more questions about my patients …’

Such engagement fosters empowerment, as it enables new nurses to build confidence, enhance critical thinking and gain a sense of control over their practice. This aligns with Empowerment Theory, which emphasises the importance of access to information, participation in decision-making and opportunities for learning as key factors in empowering individuals.

This consistent exposure to a wide variety of clinical and professional topics does more than simply enhance knowledge – it plays a pivotal role in shaping a nurse’s cognitive, emotional and professional development. Engaging with diverse discussions allows newly graduated nurses to deepen their clinical insight by encountering different perspectives, case scenarios and best practices shared by peers. This breadth of exposure facilitates more nuanced understanding and critical thinking in clinical decision-making, which not only supports clinical learning but also strengthens the psychological and professional readiness necessary for effective transition.

### Theme 2: A community of practice was formed where participants supported each other

This theme is centred on access to support and guidance from superiors and peers.

#### Professional connectedness

A community of practice provides a supportive environment where members may ask experienced nurses for advice. This encourages people to share their knowledge and learn from others, promoting lifelong learning. The participants felt encouraged and motivated when engaging in the WhatsApp group. The WhatsApp group provided them with a sense of preparedness for practice during the transition. They showed capacity to establish connections with the subjects under discussion, and the act of sharing personal experiences was beneficial in effectively navigating the challenges experienced throughout the transition process. Additionally, the graduates acquired knowledge and skills to engage effectively with colleagues through the development of good communication practices.

The outcome of professional connection was clearly revealed by the study participants. Participants were able to help one another in dealing with different and difficult situations through interaction with colleagues, which made the group serve as a pillar and made them feel equipped during transition. This was expressed by a participant who said:

‘… so I started working OBs [*Obstetrics and Gynaecology*], so at that time when we were discussing trauma, so when I went to trauma I was already equipped’. (Participant 2, 33 years, Female)

The WhatsApp group was helpful and beneficial because it provided students with guidance and support from the moderator. Participants used the group to ask for advice, which when provided, brought about the feeling of being emotionally supported, encouraged, motivated and not being alone. Additionally, peer support, through shared experiences, helped participants manage situations encountered during transition and to learn from each other’s experiences. Participants could relate to the topics of discussion:

‘… I think for me its very encouraging to know that you are not alone and if anything happens you can learn from others’ experience and advice’. (Participant 8, 31 years, Female)

#### Social connectedness

A sense of social closeness was observed among the recently graduated nurses. For example, having colleagues who worked far from each other in the group instilled a sense of positivity among the members, fostering a favourable learning experience:

‘… I felt better because, because I didn’t feel crazy, I didn’t feel as alone. I mean as you know there were people in Durban, I mean [*student’s name*] was in Durban and that made me feel a bit better about the situation …’ (Participant 4, 38 years, Male)

Individuals took advantage of the opportunity to establish connections and engage in conversations with their colleagues, and by doing so, facilitated the maintenance of social ties with friends. Participants were able to recover lost contact. Opportunity to connect and talk to colleagues helped to stay in touch and not feel alone:

‘… [*I*]t played a big role because after college [*university*], for example me, just after the comserve [*community service*], so I decided to change the number [*phone number*], on the WhatsApp, so I managed to get some people back and still form the group the one that we had in fourth year, like connection with them, we can talk to them, we can advise each other because of the group.’ (Participant 4, 38 years, Male)

### Theme 3: Moderators as catalysts of structured support and resource access

This theme captures the overarching function of moderators as catalysts who provide stability, resources and guidance in the online community. Rather than being limited to a technical role, moderators created a supportive presence that fostered accountability, problem-solving and professional confidence.

#### Online presence of moderators

The utilisation of moderators as a resource person in an online setting has proven advantageous for recently graduated nurses. Moderators, as a figure of support in the online learning environment, offer direction and assistance to novice nurses. They possess the ability to respond in a timely manner to inquiries, provide clarification on uncertainties, and provide guidance on how to effectively navigate the digital platform. This fosters a feeling of assurance and ease for novice nurses, particularly those who lack familiarity with technology or online education. In this regard, one of the participants stated that:

‘… I think your availability to answer our questions and concerns promptly … was critical at that time … [*be*]cause we might need someone to speak to immediately and other colleagues are very busy.’ (Participant 1, 42 years, Female)

Another participant stated that:

‘… I think, the fact that you are always there … you are always encouraging, like you are very persistent and you were very on to it … I don’t how to explain it, I would say that you are … not giving up, like you would always remind us and answer our questions …’ (Participant 7, 24 years, Female)

#### Moderator support and guidance on managing specific situations

The group encouraged open communication and respect among its members. However, when group members disagreed, the moderator handled it. This helped maintain a positive and happy atmosphere. Furthermore, the moderators actively promoted the development of problem-solving abilities among the participants, while also providing motivation and support to help them effectively manage and overcome challenges. A participant stated that:

‘… the moderator put every situation into scenarios which helped my problem-solving skills and the conclusions at the end of each discussion with the shared articles was educative’. (Participant 2, 33 years, Female)

Another participant stated:

‘I would say, also when you spoke about conflict management and another student would say, okay, this is what I did, and I went to the manager, because when I spoke to the nurse there was no resolution … and they had a meeting, and that is how it resolved, so I would say that is a good example.’ (Participant 7, 24 years, Female)

#### Useful materials and knowledge shared

The utilisation of various resources such as scenarios, publications, web-based links, and summaries derived from discussions can often yield advantageous outcomes. This encouraged active participation and knowledge sharing. Articles can be beneficial for recently graduated nurses, including the sharing of evidence-based knowledge on pertinent subjects such as optimal nursing methods. This was supported by participants who stated:

‘… some of the material was actually helpful, some of the attachments that were sent, most of the scenarios you can actually relate to.’ (Participant 2, 33 years, Female)

‘In many ways, especially the links, I’m not sure if it was professor [*name*] who came about with the research – helped me a lot.’ (Participant 3, 28 years, Female)

### Theme 4: Empowerment through integration of personal development and contextual relevance

Theme 4 reflects how empowerment emerged when personal skill-building was integrated with discussions that resonated directly with the realities of clinical practice. Beyond individual learning, this integration fostered confidence, self-efficacy and a stronger professional identity.

#### Development of personal skills

Personal development and acquisition of skills among the recently graduated nurses were achieved. The nurses engaged in mutual encouragement, fostering confidence, cultivating independence and enhancing their knowledge and skills, which augmented their sense of empowerment. One participant stated:

‘Yeah, it empowered me, it’s like it empowered my confidence, independence and knowledge.’ (Participant 4, 38 years, Male)

Similarly, another participant stated:

‘I would say it has empowered me in a sense that it has made me more confident going into a situation … also I feel more … competent also in some arrears … this is what you do in this situation … how do you manage the different situations, I would say it empowered, it really empowered.’ (Participant 6, 27 years, Female)

#### Discussion topics included real-life experiences

The integration of practical experiences into the discussion of the topics in the support programme served to strengthen and update the knowledge and skills obtained in an academic environment, while also promoting the development of new skills. Many topics that were discussed served to refresh their knowledge acquired throughout their education and are pertinent to their professional experiences. Participant 3 stated that the discussion topics served as a reminder of things that were taught at university and is helping to know what to do in real life situations. Another participant also mentioned:

‘… [*T*]hey were reminders of things that we already know and reminded us of those things we might have forgotten and served as a reinforcement [*or*] refresher [*or*] reminder of what was learnt in the school [*university*] and updated current skills. For example, the decision-making topic was a reminder of what I already learnt long ago, so yea it was really beneficial.’ (Participant 8, 31 years, Female)

#### Relevance of the discussion topics

The group commented on the relevance of discussion topics that empower newly graduated nurses. Several of the participants appreciated the importance of the topics discussed as it brought up several imperative themes, including the need for support and mentorship for new nurses, the importance of advocating for oneself in the workplace, and the importance of self-care and maintaining a healthy work-life balance. Most of the participants agreed that the topics were relevant. One of the participants highlighted:

‘Yes, they are relevant because it is mostly experiences of my colleagues and what they’re going through, all the ethics, yes, and being a new professional. Yes, I could relate.’ (Participant 9, 29 years, Female)

Another participant stated:

‘I think they were very relevant in the sense whereby most of the topics that were being discussed, or that we were discussing is what we see … while we are working or even as a student.’ (Participant 7, 24 years, Female)

## Discussion

This study explored the experiences of newly graduated nurses participating in a moderated WhatsApp-based peer learning intervention during their transition into professional practice. The findings indicate that structured peer engagement, facilitated by experienced moderators and grounded in real-life clinical experiences, contributed meaningfully to participants’ empowerment, professional confidence and sense of connectedness. These findings are discussed in relation to existing literature and theoretical perspectives on empowerment, communities of practice, and transition support for newly qualified nurses.

### Information sharing as a mechanism for empowerment

Access to timely, relevant and contextually meaningful information emerged as a foundational element of empowerment among newly graduated nurses. Participants described the WhatsApp group as a dynamic learning space where shared clinical scenarios, professional guidance and experiential knowledge reinforced theoretical understanding while strengthening clinical reasoning and decision-making. Rather than functioning solely as an information exchange platform, the group enabled participants to contextualise knowledge and apply it to complex clinical situations encountered in practice.

These findings are consistent with Baharum et al. (2023), who reported that peer-to-peer engagement among newly qualified nurses enhances confidence and supports professional development by providing a safe space for sharing experiences, reflection and learning. From an empowerment theory perspective, access to information and opportunities for participation are central to developing a sense of control and professional agency. The range of discussion topics, such as ethics, scope of practice, trauma management and professional conduct – enabled participants to feel better prepared for the realities of clinical practice and more confident in their professional roles.

Similarly, Gottlieb, Gottlieb and Vasiliki (2021) emphasise the importance of developing 21st-century competencies, including leadership and reflective practice, through collaborative learning environments. Continuous exposure to diverse professional topics within the WhatsApp group promoted reflective thinking and psychological readiness, which are critical during the transition period for newly graduated nurses.

### Community of practice and professional connectedness

The development of a community of practice was a significant outcome of the intervention. Professional connectedness allowed participants to seek advice, share challenges and learn from peers’ experiences, thereby reducing feelings of isolation commonly reported during the transition into professional nursing practice. Participants indicated that peer discussions enhanced their ability to manage unfamiliar clinical situations by drawing on collective knowledge and shared experiences.

These findings align with Torres et al. (2024), who highlight that strong professional networks positively influence career advancement, personal development and a sense of professional belonging. Similarly, Wei et al. (2019) found that supportive professional relationships are associated with increased career self-efficacy and job satisfaction. For newly graduated nurses, such connectedness appears particularly important during the compulsory community service period, when support structures may be limited.

Social connectedness further reinforced participants’ sense of belonging, especially for those working in geographically distant or isolated settings. Re-establishing peer relationships beyond the academic environment contributed to emotional reassurance and mutual encouragement. This is consistent with Frangieh et al. (2024), who reported that meaningful peer interactions enhance feelings of inclusion and connectedness among nursing students and graduates. The solidarity observed among participants in this study suggests that peer learning platforms can extend collegial relationships beyond formal education into early professional practice.

### Role of moderators in structured peer support

Moderators played a pivotal role in sustaining engagement, guiding discussions and maintaining psychological safety within the WhatsApp group. Their consistent online presence provided reassurance, timely feedback, and clarity when participants encountered clinical or interpersonal challenges. By framing discussions through case scenarios and reflective prompts, moderators facilitated deeper critical thinking and problem-solving.

These findings are supported by Ogolodom et al. (2023), who emphasised the importance of moderators in creating supportive and engaging online learning environments. Similarly, Kunaviktikul et al. (2022) demonstrated that effective moderation in online learning communities enhances critical thinking and problem-solving skills. The ability of moderators to manage differing opinions and foster respectful dialogue aligns with Fehrman and Watson (2021), who highlight the role of moderators in promoting constructive interaction and positive group dynamics.

The presence of experienced nurses as moderators provided participants with guidance, emotional support and access to reliable resources, which facilitated a smoother transition into professional practice. This is consistent with Liu et al. (2020) and Chuan and Ibsen (2022), who underscore the importance of ongoing education, mentorship and access to learning resources for newly graduated nurses to enhance competence and confidence.

### Integration of personal development and contextual relevance

Empowerment was further strengthened through the integration of personal skill development with discussions grounded in real-life clinical experiences. Participants reported improvements in confidence, independence, communication skills and self-efficacy. Revisiting familiar concepts from nursing education within the context of actual clinical experiences reinforced learning and supported skill renewal.

The relevance of discussion topics – such as advocacy, workplace conflict, self-care and professional boundaries – was particularly valued, as these issues reflected participants’ daily realities. Gibbs (2022) highlights that positive feedback, teamwork and opportunities for growth enhance self-efficacy among newly graduated nurses, while İspir Demir et al. (2023) link empowerment and skill acquisition to improved job satisfaction, patient care and retention.

The emphasis on real-life experiences aligns with Cowan (2014), who advocates for integrating practical experiences into academic discourse to reinforce learning. Özöztürk et al. (2023) similarly found that sharing real-life experiences promotes active engagement, reflection and critical thinking. Addressing transition-related challenges, such as workplace politics and work–life balance, further reflects findings by Zanjani, Ziaian and Ullrich (2018) and Emerson et al. (2023), who note that these issues commonly challenge newly graduated nurses during early practice.

## Conclusion

This study demonstrates that nurses in transition develop professional competence primarily through shared reflection on practice-based experiences, peer and collegial mentorship and engagement in real-world clinical settings. While newly graduated nurses often enter practice with positive expectations, these are frequently challenged by the complex realities of healthcare environments, resulting in both positive and negative transitional experiences. These perceptions are strongly shaped by the clinical context in which nurses are placed.

The findings highlight the critical need for structured and sustained support mechanisms before and after professional registration to facilitate successful transition, enhance professional confidence and promote retention within the nursing workforce. In this regard, the moderated WhatsApp-based peer learning intervention proved to be an effective support strategy, fostering empowerment, connectedness and professional learning through structured information exchange, peer support and contextually relevant discussions.

Although the study was limited to recently graduated nurses from a single university in the Western Cape, potentially restricting generalizability, the findings contribute valuable evidence supporting mobile-based peer learning as a cost-effective and accessible adjunct to formal transition programmes, particularly within resource-constrained healthcare settings.

### Limitations and further research

This study was conducted with participants from a single nursing school in the Western Cape. While the findings provide valuable insights into the transition experiences of newly graduated nurses, the limited setting may restrict the generalizability of the results. Future research should therefore include multiple institutions and diverse healthcare contexts to enhance transferability.

The use of informal digital platforms such as WhatsApp presents potential risks related to patient confidentiality, particularly when clinical experiences are discussed. In this study, these risks were mitigated through active moderation, clear guidelines for professional communication and the prohibition of sharing identifiable patient information. Nevertheless, ongoing vigilance is required when using such platforms for professional learning.

Additionally, WhatsApp lacks formal educational features, including structured content organisation, assessment tools, progress tracking and modular delivery. As a result, evaluating learning engagement and outcomes within a WhatsApp-based intervention is inherently challenging because of the absence of built-in analytics to monitor participation or measure learning impact.

Finally, the study did not assess potential confounding factors, such as other support programmes offered as part of the compulsory community service programme, which may have influenced the outcomes and limited the making of causal inferences about the effects of the intervention.
